# Surveillance of Phenibut in Wastewater During a Brazilian Carnival

**DOI:** 10.1002/dta.70002

**Published:** 2025-11-23

**Authors:** Bruna R. de S. Gomes, Ana Flávia B. de Oliveira, Aline de Melo Vieira, Dhayaalini Nadarajan, Richard Bade, Jandyson M. Santos

**Affiliations:** ^1^ Petroleum, Energy and Mass Spectrometry Research Group, Chemistry Department Universidade Federal Rural de Pernambuco, UFRPE Recife Pernambuco Brazil; ^2^ Queensland Alliance for Environmental Health Sciences (QAEHS) University of Queensland Woolloongabba Queensland Australia

**Keywords:** Brazilian carnival, new psychoactive substances, phenibut, wastewater‐based epidemiology

## Abstract

Phenibut is a new psychoactive substance (NPS) first synthesized in Russia in 1963 as a derivative of gamma‐aminobutyric acid. Originally developed for therapeutic use, it has gained popularity for nonmedical purposes, including recreational and cognitive enhancement. In Brazil, phenibut is uncontrolled and easily purchased online. This study used wastewater‐based epidemiology (WBE) to investigate phenibut use patterns in two northeastern Brazilian cities. Composite daily wastewater samples were collected from two treatment plants (WWTPs), Recife (WWTP_A_) and Olinda (WWTP_B_), during two periods in 2023: Carnival and a reference week. Samples underwent solid‐phase extraction (SPE) and analysis by liquid chromatography–tandem mass spectrometry (LC–MS/MS). Phenibut concentrations were converted to population‐normalized mass loads (PNMLs, mg/day/1000 inhabitants). The highest phenibut levels and PNMLs (up to 4.06 mg/day/1000 inhabitants) occurred during Carnival at WWTP_A_, located in a major tourist area, suggesting recreational use. During the reference week, PNMLs ranged from detection limits to 2.29 mg/day/1000 inhabitants on weekdays, indicating possible functional or cognitive enhancement use. These findings reveal two distinct use patterns: recreational peaks during Carnival weekends and possible functional use on weekdays outside festive periods. This is the first evidence of phenibut detection in Brazilian wastewater and its temporal use patterns. The results highlight WBE's value in monitoring NPS trends and suggest recreational use predominates during large events. This underscores the need for public health attention and regulatory monitoring of uncontrolled substances with abuse potential.

## Graphical abstract

This study reports the first detection of phenibut in Brazilian wastewater. Phenibut, a substance readily sold online to improve concentration, reaches wastewater treatment plants (WWTPs) after consumption by the population. Samples from WWTPs in the cities of Recife and Olinda in Northeastern Brazil were collected during the world's largest Carnival and analyzed by LC–MS/MS after solid‐phase extraction (SPE). Mass loading levels (mg/day/1000 inhabitants) were higher during Carnival than during the reference week, suggesting possible recreational use during the festive period.

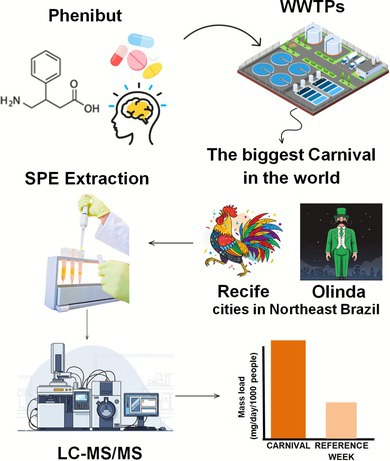

## 1 Introduction

In recent decades, a new class of substances known as new psychoactive substances (NPSs) has attracted increasing attention in the fields of public health and safety [1]. NPSs are compounds designed to mimic the effects of controlled drugs and often circumvent existing drug legislation through slight structural modifications. These substances may be synthetic or derived from plant sources, and their growing availability and diversity present significant challenges for regulation, detection, and public health response [2].

Over the years, numerous NPS have emerged. One such substance is phenibut, originally synthesized in Russia in 1963, which has been used clinically for anxiety, insomnia, post‐traumatic stress disorder, and alcohol withdrawal. Acting on both GABA_A_ and GABA_B_ receptors, it is marketed as a nootropic agent [3]. While it is a controlled substance in some countries, such as Hungary, Italy, Lithuania, France, and Australia [4], it remains unregulated in most parts of the world, including Brazil.

Although some clinical reports exist, data on phenibut's effects, toxicity, and withdrawal remain limited, raising concerns due to its easy online availability [5, 6]. Unsupervised use is increasing, with cases of acute intoxication, including instances requiring mechanical ventilation when combined with ethanol [7], as well as reports of severe dependence characterized by high intake and withdrawal symptoms [8]. Online accounts describe its use as a substitute for illicit drugs, to boost alcohol effects, or to mask intoxication [7].

Traditional methods for estimating drug use, such as self‐reported surveys, toxicology tests, and criminal statistics, have limitations in terms of comprehensiveness, accuracy, and timeliness. In this context, wastewater‐based epidemiology (WBE) emerges as a noninvasive, real‐time, and cost‐effective tool capable of estimating NPS use at low concentrations. The first detection of phenibut in wastewater was reported in 2024 [9], in samples collected from a wastewater treatment plant (WWTP) in Cyprus, a Mediterranean country. Subsequently, phenibut was also identified in Australian capital cities [10, 11]. These findings underscore the need for environmental monitoring of phenibut, particularly in regions where regulatory control is lacking.

To the best of our knowledge, no studies have yet investigated the presence of phenibut in environmental matrices or documented consumption patterns in Brazil. This lack of data, combined with easy online access and the absence of regulation, highlights the urgency of implementing monitoring efforts. Therefore, there is a pressing need to expand the monitoring and investigation of phenibut occurrence, particularly in regions where its use is not regulated, such as Brazil. In this study, we monitored phenibut in raw wastewater samples collected during one of the largest popular festivals in the world, the 2023 Carnival, and during a reference week in Pernambuco State, Northeast Brazil, to assess its presence and potential recreational use.

## 2 Methods

### 2.1 Study Area, Samples, and Extraction

**FIGURE 1:**
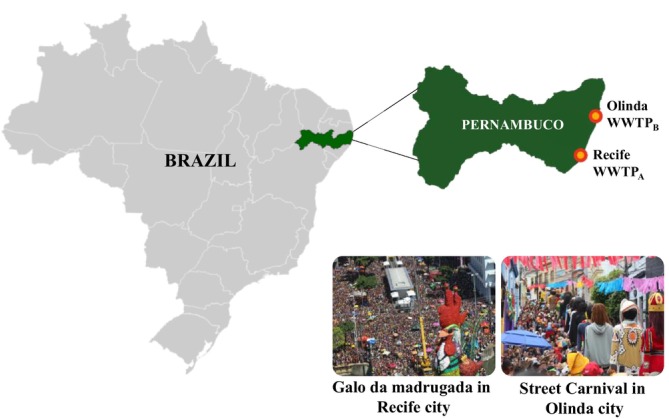
Representative map showing the location of sample collection points in Recife (WWTP_A_) and Olinda (WWTP_B_) cities, Pernambuco state (northeast Brazil), during Carnival (February 17–23, 2023) and a reference week (August 30–September 5, 2024).

Raw wastewater samples were collected from WWTPs, coded as WWTP_A_ (Recife city) and WWTP_B_ (Olinda city), over two distinct 7‐day periods: Carnival (February 17–23, 2023) and a reference week (August 30–September 5, 2024), totaling 28 samples (Figure 1). WWTP_A_ receives wastewater from the main municipalities of Recife, including the area hosting the “Galo da Madrugada” parade, the largest annual Carnival event in Recife [12]. In turn, WWTP_B_ covers the historic city of Olinda, famous for its traditional street festivities. The weekly population size in the two collection periods was estimated based on measurements of ammonium nitrogen (NH₄‐N) using the phenate method, as described in the *Standard Methods for the Examination of Water and Wastewater* [13] (Supplementary Materials, Section 1).

During the sampling period, 1‐L grab samples of untreated wastewater were manually collected from the two WWTPs between 8:00 and 10:00 a.m., before treatment, on 7 days. After collection, the samples were transferred to amber glass bottles that had been prewashed with water, 5% Extran (Merck KGaA, Darmstadt, Germany), ultrapure water, and ethanol, and were then transported to the laboratory for processing. Sample preparation based on SPE was performed as described by [14] and is detailed in the Supplementary Material in Section 2.

### 2.2 LC–MS/MS Analysis

The LC–MS/MS analysis was performed, and is detailed in the Supplementary Material, Section 3. See MRM transitions in Table S1 and validation parameters in Table S2.

### 2.3 Phenibut Consumption

The daily load of phenibut was calculated by multiplying the measured phenibut concentration (ng L^−1^) by the daily influent flow rate (L day^−1^), according to Equation (1):

(Eq 1) $$\text{Load}\;\left(g/\mathrm{day}\right)=\text{Phenibut concentration}\;\left(\frac{\mathrm{ng}}{L}\right)\times \text{flow rate}\;\left(L\;{\mathrm{day}}^{-1}\right)\times 10^{-9}$$

The population‐normalized mass load (PNML) of phenibut was estimated by dividing the daily load by the population size of each WWTP, normalized to 1000 inhabitants, as described in Equation (2). The population served was estimated based on ammonium nitrogen (NH₄‐N) concentrations (see Section 1 of Supplementary Material). Because of the lack of pharmacokinetic data, no correction for the parent/metabolite ratio was applied.

(Eq 2) $$\text{PNML}\;\left(\mathrm{mg}/\mathrm{day}/1000\;\text{inhabitants}\right)=\text{Load}\;\left(g/\mathrm{day}\right)\times \frac{1000}{\text{population}}\times 10^{-6}$$

### 2.4 Statistical Analysis

The normality of PNML values was assessed using the Shapiro–Wilk test, which indicated a nonnormal distribution. Because of this violation of the normality assumption, values below the limit of detection (LOD) were replaced with LOD/2 to avoid zero values. Subsequently, an aligned rank transform (ART) ANOVA was applied to analyze PNML values. The analysis included comparisons of phenibut data among treatment plants (WWTP_A_ vs. WWTP_B_), sampling periods (Carnival vs. reference week), and day types (weekdays vs. weekends), as well as interactions among these factors. All statistical analyses were conducted using RStudio (Version 2024.09.1) with the ARTool package and other relevant libraries.

## 3 Results

**FIGURE 2:**
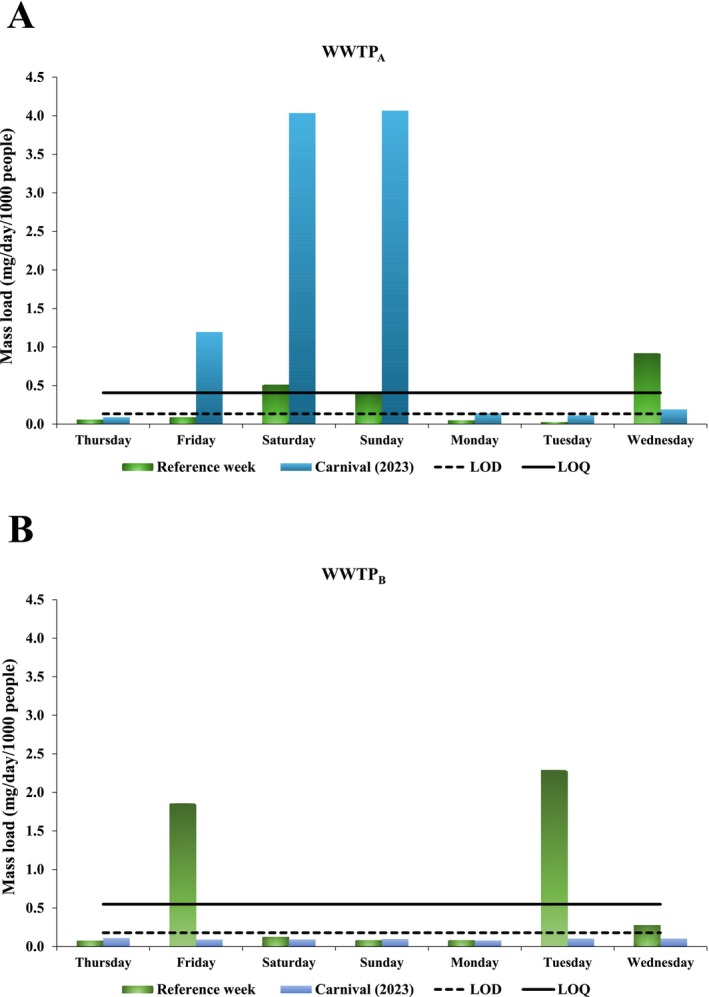
Population‐normalized mass load (mg/day/1000 inhabitants) of phenibut in waste‐water samples based on concentrations measured for the community served by WWTP_A_ (A) and WWTP_B_ (B), during a reference week and 2023 Carnival.

The LC–MS/MS chromatograms of phenibut and the internal standard MDMA‐d_5_ at a calibration level of 250 ng L^−1^ are shown in Figure S1. In samples, phenibut concentrations ranged from < LOD to 41.9 ng L^−1^ in WWTP_A_, and from < LOD to 14.3 ng L^−1^ in WWTP_B_ (Tables S3 and S4) for all samples in both periods. The LODs were 0.13 and 0.18 ng L^−1^ for WWTP_A_ and WWTP_B_, respectively. Note that great variability was found when comparing the concentrations, with the highest values observed in the Carnival samples (Table S4). To better understand, PNML was calculated, as presented in Tables S5 and S6 and graphically illustrated in Figure 2.

The mass load values obtained in WWTP_A_ ranged from < LOD to 0.92 mg/day/1000 inhabitants during the reference week, and from < LOD to 4.06 mg/day/1000 inhabitants during the Carnival period, which means approximately four times more during the festival. The values obtained in WWTP_B_ ranged from < LOD to 2.29 mg/day/1000 inhabitants during the reference week and were below the LOD during Carnival.

**FIGURE 3:**
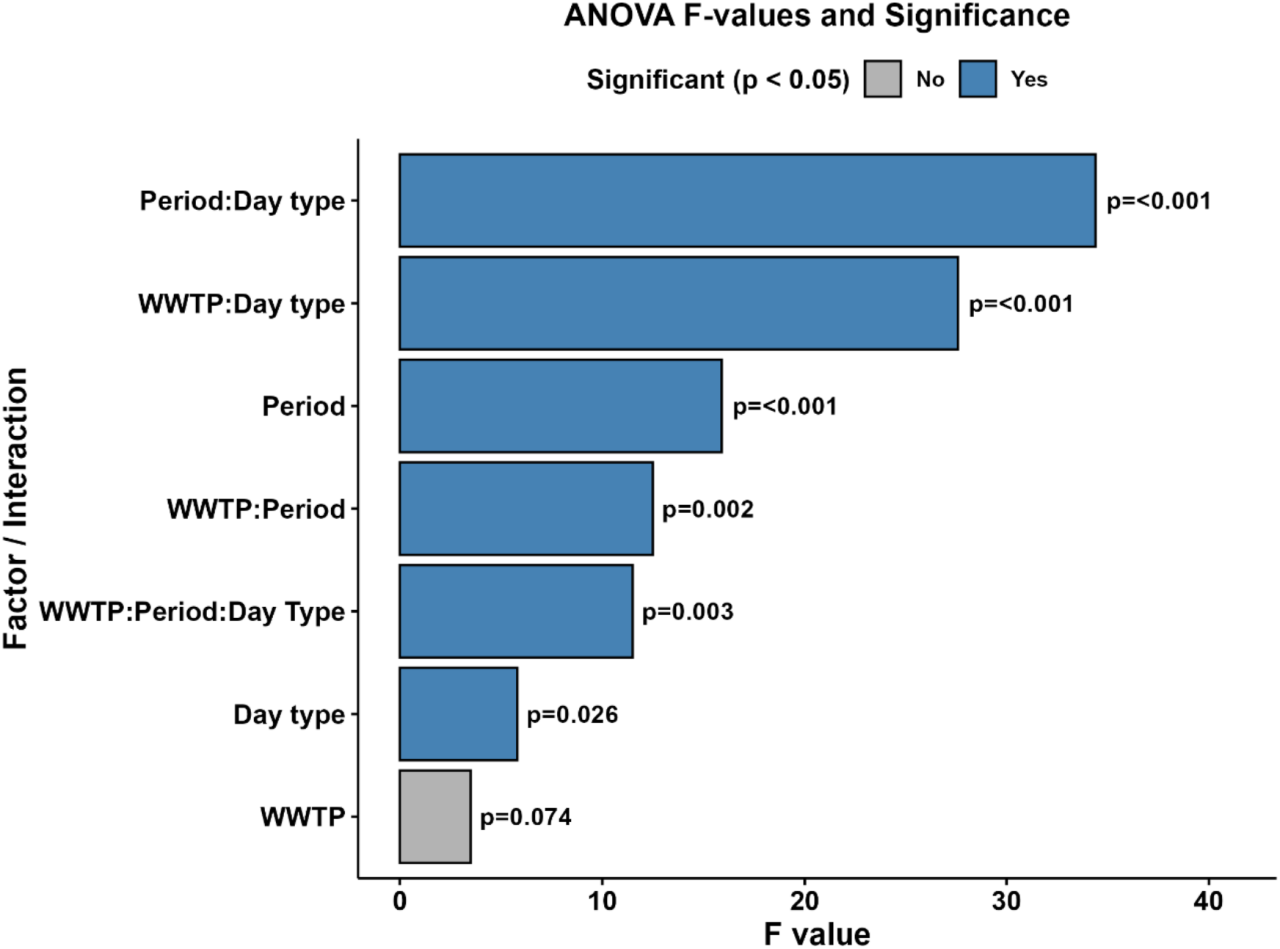
Results of the ART ANOVA showing *F* values and *p* values for nonparametric factorial effects. Significant effects (*p* < 0.05) are highlighted in blue.

The Shapiro–Wilk test indicated nonnormality for PNML values (*W* = 0.5847; *p* < 0.001), although Levene's test confirmed homogeneity of variances (*F* (7, 20) = 1.2142; *p* = 0.3403), according to Table S7. Therefore, an ART ANOVA was performed, showing significant effects for Period (*p* = 0.0007) and Day type (weekday vs. weekend; *p* = 0.0257), while the effect of WWTP (WWTP_A_ vs. WWTP_B_) showed a marginal trend (*p* = 0.074). Significant interactions were observed among WWTP: Period (*p* = 0.0021), WWTP: Day type (*p* < 0.0001), Period: Day type (p < 0.0001), and the three‐way interaction WWTP: Period: Day type (*p* = 0.0029). These results are illustrated in Figure 3 and detailed in Table S8.

## 4 Discussion

The highest PNML values (Figure 2) were recorded during weekend sampling at WWTP_A_, specifically during the Carnival festivities. The maximum value was observed on Saturday (February 18, 2023), the date of the “Galo da Madrugada” parade, which is considered the day with the highest concentration of revelers and the largest audience during Recife's Carnival. These findings suggest potential recreational use of phenibut, associated with the elevated levels detected during the festive period.

It is interesting to note that phenibut was detected only in samples collected at WWTP_A_ during Carnival, with the highest PNML values reaching 1.19, 4.04, and 4.06 mg/day/1000 inhabitants on Friday, Saturday, and Sunday, respectively (Figure 2). This can be explained by socio‐economic and geographic differences between the areas served by the treatment plants. WWTP_A_ is located in the state capital, serves an area that hosts tourists during the carnival festival, and likely has greater availability of the drug, factors that may have contributed to increased substance use. The visitor influx during the 2023 Carnival is reflected by a 96.12% hotel occupancy rate [15] in the region of the city where WWTP_A_ is located.

It is worth noting that the estimated population contributing to the WWTPs during Carnival is similar to that of the reference week. Although Carnival attracts a larger number of people to the city, many attendees use portable toilets and other temporary sanitation facilities in the festival area, which are not connected to the sewage system. As a result, part of the wastewater is diverted and does not reach the WWTPs. However, phenibut concentrations were notably higher during Carnival, which could mean increased or more frequent consumption of phenibut.

During the reference week, PNML values were subtly higher on weekdays (Figure 2). This pattern may suggest the use of the substance in contexts related to academic or professional activities, possibly due to its reported nootropic effects. It is worth noting that several public service examinations were held across the city during this period, which may be related to the use by individuals preparing for these competitive exams [16].

The Shapiro–Wilk test (Table S7) indicated that phenibut concentration values did not follow a normal distribution (*W* = 0.5847, *p* < 0.001). Because PNML values are derived from these concentrations, their residuals also violated the normality assumption (*W* = 0.8289, *p* = 0.00036), despite Levene's test confirming homogeneity of variances (*F* (7, 20) = 1.2142, *p* = 0.3403).

Therefore, a nonparametric approach using the ART was applied (Table S8; Figure 3), revealing significant main effects for Period (Carnival vs. reference week; *p* = 0.0007) and Day type (weekday vs. weekend; *p* = 0.0257). Interaction effects were observed between WWTP and Period (*p* = 0.0021), WWTP and Day type (*p* < 0.0001), Period and Day type (p < 0.0001), and the three‐way interaction WWTP × Period × Day type (*p* = 0.0029). These findings highlight how phenibut consumption varies across locations, periods, and types of days, reinforcing the importance of temporal and spatial factors in monitoring emerging substances.

The highest phenibut concentrations detected during Carnival days in Brazil reflect a global pattern, where consumption increases during major social events. Previous reports from Cyprus and Australia have also shown peaks of phenibut during festivals and large gatherings [10, 11], indicating probable recreational use during these periods. This study is limited to data from two WWTPs and may not fully represent phenibut use nationwide. Broader monitoring is necessary to gain a better understanding of national consumption patterns.

## 5 Conclusions

This study provides the first evidence of the occurrence and quantification of phenibut in Brazilian wastewater. Consumption was detected during the 2023 Carnival (the largest festive event in Brazil) and in a reference week. We found two distinct patterns of phenibut use, as follows: (1) The highest levels were found in samples from the Carnival period, with peak concentrations occurring on weekends, suggesting recreational use in festive contexts, and (2) during nonfestive periods, the highest levels were observed on weekdays, possibly related to therapeutic use for enhancing concentration and cognitive performance. Thus, the detection of phenibut in these different contexts underscores the importance of analyzing temporal dynamics in the surveillance of NPS, and suggests that in Brazil, it has been consumed mainly recreationally, with potential for abuse.

## Author Contributions

**Bruna R. de S. Gomes:** conceptualization, methodology, investigation and writing – original draft. **Ana Flávia B. de Oliveira:** conceptualization, methodology, investigation, writing – review and editing. **Aline de Melo Vieira:** methodology, investigation, writing – review and editing. **Dhayaalini Nadarajan:** methodology, formal analysis, investigation, writing – review and editing. **Richard Bade:** methodology, formal analysis, investigation, resources, writing – review and editing. **Jandyson M. Santos:** supervision, methodology, investigation, resources, writing – review and editing.

## Funding

This work was supported by the Coordenação de Aperfeiçoamento de Pessoal de Nível Superior (AUXPE 88881.993362/2024‐01, RENENSP Project) and Australian Research Council Discovery Early Career Award (Project Number DE220100381).

## Conflicts of Interest

The authors declare no conflicts of interest.

## Supporting information


**Figure S1:** LC–MS/MS chromatograms of phenibut and the internal standard MDMA‐d_5_ at a calibration level of 250 ng L^−1^.
**Table S1:** MRM transitions and MS parameters for phenibut and the internal standard.
**Table S2:** Validation parameters of the LC–MS/MS method for phenibut in wastewater samples.
**Table S3:** Phenibut concentration (ng L^−1^), flow rate (L day^−1^), total mass (ng), and load (g day^−1^) for the reference week in WWTP_A_ and WWTP_B_.
**Table S4:** Phenibut concentration (ng L^−1^), flow rate (L day^−1^), total mass (ng), and load (g day^−1^) for 2023 Carnival in WWTP_A_ and WWTP_B_.
**Table S5:** Results of the reference week for the WWTP_A_ and WWTP_B_ stations, showing the PNML (mg/day/1000 inhabitants).
**Table S6:** Results of the 2023 Carnival days for the WWTP_A_ and WWTP_B_ stations, showing the PNML (mg/day/1000 inhabitants).
**Table S7:** Results of the Shapiro–Wilk test for normality of PNML data and Levene's test for homogeneity of variances. The Shapiro–Wilk test indicates a significant departure from normality (*p* < 0.05), while Levene's test suggests homogeneity of variances across groups (*p* > 0.05).
**Table S8:** Results of the ART ANOVA showing *F* values and *p* values for nonparametric factorial effects.

## Data Availability

The data that supports the findings of this study are available in the supplementary material of this article.
