# Supplementary material for: Surveillance of Phenibut in Wastewater During a Brazilian Carnival
Source: Drug Test Anal. 2025 Nov 23;18(2):192–7. doi: 10.1002/dta.70002 (PMC12861594; doi:10.1002/dta.70002)
Supplement: Supplementary file 1 — Figure S1: LC–MS/MS chromatograms of phenibut and the internal standard MDMA‐d5 at a calibration level of 250 ng L−1. Table S1: MRM transitions and MS parameters for phenibut and the internal standard. Table S2: Validation parameters of the LC–MS/MS method for phenibut in wastewater samples. Table S3: Phenibut concentration (ng L−1), flow rate (L day−1), total mass (ng), and load (g day−1) for the reference week in WWTPA and WWTPB. Table S4: Phenibut concentration (ng L−1), flow rate (L day−1), total mass (ng), and load (g day−1) for 2023 Carnival in WWTPA and WWTPB. Table S5: Results of the reference week for the WWTPA and WWTPB stations, showing the PNML (mg/day/1000 inhabitants). Table S6: Results of the 2023 Carnival days for the WWTPA and WWTPB stations, showing the PNML (mg/day/1000 inhabitants). Table S7: Results of the Shapiro–Wilk test for normality of PNML data and Levene's test for homogeneity of variances. The Shapiro–Wilk test indicates a significant departure from normality (p < 0.05), while Levene's test suggests homogeneity of variances across groups (p > 0.05). Table S8: Results of the ART ANOVA showing F values and p values for nonparametric factorial effects. [file DTA-18-192-s001.docx]

**SUPPLEMENTARY MATERIAL**

SURVEILLANCE OF PHENIBUT IN WASTEWATER DURING A BRAZILIAN CARNIVAL

**Bruna R. de S. Gomes^a^, Ana Flávia B. de Oliveira^a^, Aline de Melo Vieira^a^, Dhayaalini Nadarajan^b^, Richard Bade^b^, Jandyson M. Santos^a^***

*^a^Petroleum, Energy and Mass Spectrometry Research Group, Chemistry Department, Universidade Federal Rural de Pernambuco, UFRPE, 52171-900 Recife – PE, Brazil*

*^b^Queensland Alliance for Environmental Health Sciences (QAEHS), The University of Queensland, 20 Cornwall Street, Woolloongabba, Queensland 4102, Australia*

^*^Corresponding author: E-mail: jandyson.machado@ufrpe.br (Santos, J. M.); ORCID: 0000-0002-2099-6728

1. **Estimation of Population Size**

The daily population served by each wastewater treatment plant (WWTP) was estimated based on ammonium nitrogen (NH_4_^+^-N) concentrations, determined using the phenate method ^[1]^. Absorbance was measured at 640 nm with a UV-Vis spectrophotometer (UV-5100, Shanghai Metash). A calibration curve (0.01–1.0 mg L^-1^, dilution factor 100) was applied. Population (P) was calculated using Equation (1):

P = $\frac{C_{n^{x}}Q_{j}}{R_{n}}$ Eq.1

where *Cn* is the NH₄⁺-N concentration (mg L^-1^), *Qj* is the daily flow (L day^-1^), and *Rn* is the per capita NH₄⁺-N contribution (6,900 mg day^-1^) ^[2,3]^.

In both WWTPs, NH₄⁺-N concentrations were lower during Carnival compared to a reference week, likely due to the use of portable toilets and temporary population shifts. These trends align with previous studies reporting reductions in NH₄⁺-N levels and estimated population size during holidays and large events ^[4]^. WWTP_A_ had a weekly estimated population of 190,211.67 during the reference week, and 128,604.65 during the Carnival period; WWTP_B_ served an estimated weekly population of 197,005.34 and 135,084.926 inhabitants during the reference week and Carnival, respectively. The reduction in the population contributing to the sewage system during Carnival could reflect the use of alternative waste disposal methods, such as portable toilets and public urination, which bypass the municipal sewage infrastructure. Thus, although the total number of people in the city increased during Carnival, a smaller proportion of the population may contribute to the wastewater collected at the WWTPs.

1. **Extraction procedure**

At the time of collection, all wastewater samples (100 mL) were immediately transported in coolers under refrigeration (4 °C). On the same day, the samples were homogenized, and 100 mL aliquots were centrifuged at 5000 rpm for 5 min. The supernatants were vacuum-filtered using GF/A glass microfiber filters (1.6 µm, Whatman, UK) and acidified to pH 2. After filtration, the pH was adjusted between 4.5 and 5.0 by adding 28% aqueous ammonia (Vetec, Rio de Janeiro, Brazil). The internal standard (±)-3,4-Methylenedioxymethamphetamine-d_5_ (MDMA-d_5_) (Cerilliant, Round Rock, TX, USA) was subsequently added to each sample, which was gently mixed to ensure homogeneity.

Solid Phase Extraction (SPE) was then performed using UCT XtracT DAU cartridges, which combine cation-exchange and hydrophobic properties, as described by ^[5]^. Before sample loading, the SPE cartridges were conditioned sequentially with 6 mL of methanol (Supelco, Pennsylvania, USA) and 6 mL of 20 mM sodium acetate buffer (Química Moderna, Minas Gerais, Brazil) (pH 5). The prepared wastewater samples were loaded by gravity until they were fully loaded. After washing with 6 mL of sodium acetate buffer, 2 mL of 0.1 mol L^-1^ acetic acid (Química Moderna, Minas Gerais, Brazil), and 6 mL of methanol, the cartridges were air-dried under vacuum for 15 min to prevent degradation in the aqueous matrix.

Drying the SPE cartridges minimizes hydrolytic and biological degradation because the analytes remain adsorbed on the sorbent rather than in solution. The dried cartridges were stored at −20 °C and transported to the University of Queensland for elution and LC–MS/MS analysis. Under these dry and refrigerated conditions, analyte losses are negligible. Previous on-cartridge stability assessments have shown less than 20% loss of phenibut at −20 °C for up to two weeks of storage ^[6]^.

Upon arrival at the University of Queensland, analytes were eluted from the SPE cartridges with a solvent mixture containing 80% dichlorometahane,16% isopropanol, and 4% ammonia (28%) (Fisher Scientific, Tingalpa, QLD, Australia). The extracts were analyzed by LC-MS/MS.

1. **Method Validation and LC-MS/MS analysis**

SPE extracts were quantified using liquid chromatography-tandem mass spectrometry (LC-MS/MS), as previously described by ^[5]^, utilizing a Shimadzu Nexera LC-40 (Kyoto, Japan) coupled to a SCIEX Triple Quad 7500 system (Framingham, MA, USA). Chromatographic separation was performed using a Phenomenex Kinetex Biphenyl column (50 mm x 2.1 mm, 2.6 µm) fitted with Phenomenex Security Guard Ultra cartridges HPLC Biphenyl 2.1 mm ID columns at a flow rate of 0.45 mL min^-1^ and an injection volume of 2.0 μL. The column oven temperature was maintained at 40 °C ± 1 °C, and the autosampler was kept between 4 °C and 7 °C. A C18 delay column was (50 x 2.1 mm, 3 µm) was installed between the LC outlet and the MS source. Mobile phase A consisted of 95% ultrapure water (Millipore, Bedford, USA) with 5% MeOH (Merck Pty Ltd, Victoria, Australia) and 0.1% formic acid (Sigma-Aldrich Pty Ltd, Castle Hill, Australia), while mobile phase B consisted of 95% MeOH with 5% ultrapure water and 0.1% formic acid. The chromatographic gradient was as follows: 5% B at 0 min held for 1 min, linear increase to 100% B at 7 min held for 2 min, then decreased to 5% B at 9.1 min and kept steady for the final 1.9 min to equilibrate the system. The total runtime was 11 min.

The mass spectrometer was run in scheduled multiple reaction monitoring (sMRM) mode in positive ionization mode, with a 30 s retention time window around the expected retention time of 0.84 min for Transition 1 (T1) at *m/z* 180.1 → 145.0 and Transition 2 (T2) at *m/z* 180.1 → 117.0. The source temperature was set at 480 °C, the ion spray voltage was 2000 V, GS1 (nebulizer gas) was 50 psi, and GS2 (heating gas) was 80 psi; curtain gas and collision gas (CAD) were set to 40 psi and 9 psi, respectively. The optimized exit potential (EP), collision energy (CE, T1/T2), collision cell exit potential (CXP, T1/T2), and Q0 dissociation were 10 V, 20 V / 25 V, 15 V / 15 V, and 4.0 V. The MRM transitions, retention times, and optimized MS parameters for phenibut and the internal standard (MDMA-d_5_) are provided in Table S1. LC-MS/MS chromatograms for phenibut and MDMA-d_5_ at the calibration concentration of 250 ng L^-1^ are provided in Figure S1.

The limit of quantification was 3.3 ng L^-1^, the linearity range was between 3.3 and 500 ng L⁻¹ with an R² of 0.9940, the extraction recovery at 10 ng L^-1^ was 41%, with a matrix effect of 151% ^[7]^. A summary of the main validation parameters, including LOD, LOQ, linearity, recovery, matrix effects, and precision, is provided in Table S2.

**Figure S1.** LC-MS/MS chromatograms of phenibut and the internal standard MDMA-d_5_ at a calibration level of 250 ng L^-1^.

**Table S1.** MRM transitions and MS parameters for phenibut and the internal standard.

| Compound | Rt (min) | Precursor Ion  (*m/z*) | Product Ion 1  (*m/z*) | Product Ion 2  (*m/z*) | EP  (V) | CE 1  (V) | CE 2  (V) | CXP 1  (V) | CXP 2  (V) | Q0D  (V) |
| --- | --- | --- | --- | --- | --- | --- | --- | --- | --- | --- |
| Phenibut | 1.24 | 180.1 | 145.0 | 117.0 | 10.0 | 20 | 25 | 15 | 15 | 4.0 |
| IS (MDMA-d5) | 3.65 | 199.2 | 165.1 | - | 10 | 17 | - | 16 | - | 5.7 |

**Table S2.** Validation parameters of the LC–MS/MS method for phenibut in wastewater samples.

| NPS compound | LOD (ng L^-1^) | LOQ (ng L^-1^) | Linearity range (ng L^-1^) | (R^2^) | ER at 10 (ng L^-1^) | ME (%) at 10 (ng L^-1^) | Intra-day repeatability at 10 ng L^-1^ [% RSD] | Intra-day repeatability at 100 ng L^-1^ [% RSD] |
| --- | --- | --- | --- | --- | --- | --- | --- | --- |
| Phenibut | 1.1 | 3.3 | LOQ−500 | 0.9940 | 41 | 151 | 13 | 12 |

**Table S3.** Phenibut concentration (ng L^-1^), flow rate (L day^-1^), total mass (ng), and load (g day^-1^) for the reference week in WWTP_A_ and WWTP_B_.

|  |  | WWTP_A_ |  |  |
| --- | --- | --- | --- | --- |
| REFERENCE WEEK | **Concentration**  **(ng L^-1^)** | **Flow rate**  **(L day^-1^)** | **Total mass**  **(ng)** | **Load**  **(g day^-1^)** |
| Thursday | <LOD | 20,154,528 | <LOD | <LOD |
| Friday | 1.2 | 13,872,384 | 16,819,913.728 | 0.017 |
| Saturday | 5.9 | 16,441,056 | 97,352,435.398 | 0.097 |
| Sunday | 7.2 | 10,546,848 | 75,826,727.576 | 0.076 |
| Monday | <LOD | 16,248,384 | <LOD | <LOD |
| Tuesday | <LOD | 9,939,456 | <LOD | <LOD |
| Wednesday | 8.8 | 19,827,072 | 174,921,832.666 | 0.175 |
|  |  | **WWTP_B_** |  |  |
| Thursday | <LOD | 27,954,720 | <LOD | <LOD |
| Friday | 11.8 | 30,994,272 | 365,993,121.388 | 0.366 |
| Saturday | 0.8 | 30,856,032 | 24,473,032.731 | 0.024 |
| Sunday | <LOD | 30,115,584 | <LOD | <LOD |
| Monday | <LOD | 29,561,760 | <LOD | <LOD |
| Tuesday | 14.3 | 31,523,040 | 450,999,142.864 | 0.451 |
| Wednesday | 2.1 | 26,191,296 | 54,590,738.863 | 0.054 |

**Table S4.** Phenibut concentration (ng L^-1^), flow rate (L day^-1^), total mass (ng), and load (g day^-1^) for 2023 Carnival in WWTP_A_ and WWTP_B_.

| CARNIVAL | Concentration  (ng L^-1^) | Flow rate  (L day^-1^) | Total mass  (ng) | Load  (g day^-1^) |
| --- | --- | --- | --- | --- |
| WWTP_A_ | | | | |
| Thursday | <LOD | 21,090,240 | <LOD | <LOD |
| Friday | 14.4 | 10,632,384 | 153,548,069.498 | 0.153 |
| Saturday | 28.7 | 18,067,104 | 518,786,091.991 | 0.519 |
| Sunday | 41.9 | 12,480,480 | 522,857,214.112 | 0.523 |
| Monday | <LOD | 33,472,224 | <LOD | <LOD |
| Tuesday | <LOD | 26,901,504 | <LOD | <LOD |
| Wednesday | <LOD | 45,219,168 | <LOD | <LOD |
|  |  | **WWTP_B_** |  |  |
| Thursday | <LOD | 27,220,320 | <LOD | <LOD |
| Friday | <LOD | 22,342,176 | <LOD | <LOD |
| Saturday | <LOD | 22,693,824 | <LOD | <LOD |
| Sunday | <LOD | 24,458,976 | <LOD | <LOD |
| Monday | <LOD | 19,402,848 | <LOD | <LOD |
| Tuesday | <LOD | 25,736,832 | <LOD | <LOD |
| Wednesday | <LOD | 36,472,032 | <LOD | <LOD |

**Table S5.** Results of the reference week for the WWTP_A_ and WWTP_B_ stations, showing the PNML (mg/day/1000 inhabitants).

| REFERENCE WEEK | PNML  (mg/day/1000 inhabitants) |
| --- | --- |
| WWTP_A_ | |
| Thursday | <LOD |
| Friday | 0.09 |
| Saturday | 0.51 |
| Sunday | 0.40 |
| Monday | <LOD |
| Tuesday | <LOD |
| Wednesday | 0.92 |
| WWTP_B_ | |
| Thursday | <LOD |
| Friday | 1.86 |
| Saturday | 0.12 |
| Sunday | <LOD |
| Monday | <LOD |
| Tuesday | 2.29 |
| Wednesday | 0.28 |

**Table S6.** Results of the 2023 Carnival days for the WWTP_A_ and WWTP_B_ stations, showing the PNML (mg/day/1000 inhabitants).

| CARNIVAL | PNML  (mg/day/1000 inhabitants) |
| --- | --- |
| WWTP_A_ | |
| Thursday | <LOD |
| Friday | 1.19 |
| Saturday | 4.04 |
| Sunday | 4.06 |
| Monday | <LOD |
| Tuesday | <LOD |
| Wednesday | <LOD |
| WWTP_B_ | |
| Thursday | <LOD |
| Friday | <LOD |
| Saturday | <LOD |
| Sunday | <LOD |
| Monday | <LOD |
| Tuesday | <LOD |
| Wednesday | <LOD |

**Table S7.** Results of the Shapiro-Wilk test for normality of PNML data and Levene’s test for homogeneity of variances. The Shapiro-Wilk test indicates a significant departure from normality (p < 0.05), while Levene’s test suggests homogeneity of variances across groups (p > 0.05).

| Test | Statistic | p-value | Degrees of Freedom  (if applicable) | Interpretation |
| --- | --- | --- | --- | --- |
| Shapiro-Wilk | W = 0.5847 | 9.599x10^-8^ | – | Non-normal distribution |
| Levene’s Test | \|  \| \| --- \|   F = 1.2142 | 3.403x10^-1^ | D_f_ = 7; 20 | Homogeneity of variances assumed (p > 0.05) |

**Table S8.** Results of the ART ANOVA showing F-values and p-values for nonparametric factorial effects.

| Factor | F_value | p_value | Significant | p_label |
| --- | --- | --- | --- | --- |
| **WWTP** | 3.5 | 0.0740 | No | 0.074 |
| **Period** | 15.9 | 0.0007 | Yes | <0.001 |
| **Day type** | 5.8 | 0.0257 | Yes | 0.026 |
| **WWTP:Period** | 12.5 | 0.0021 | Yes | 0.002 |
| **WWTP:Day type** | 27.6 | 3.8x10^-5^ | Yes | <0.001 |
| **Period:Day type** | 34.4 | 9.0x10^-6^ | Yes | <0.001 |
| **WWTP:Period:Day Type** | 11.5 | 0.0029 | Yes | 0.003 |

**4. REFERENCES**

1. Rice EW, Baird RB, Eaton AD. Standard Methods for the Examination of Water and Wastewater, 23rd Edition.

2. Been F, Rossi L, Ort C, Rudaz S, Delémont O, Esseiva P. Population normalization with ammonium in wastewater-based epidemiology: Application to illicit drug monitoring. Environ Sci Technol [Internet]. American Chemical Society; 2014 [cited 2025 May 28];48:8162–9. https://doi.org/10.1021/ES5008388

3. Croft TL, Huffines RA, Pathak M, Subedi B. Prevalence of illicit and prescribed neuropsychiatric drugs in three communities in Kentucky using wastewater-based epidemiology and Monte Carlo simulation for the estimation of associated uncertainties. J Hazard Mater [Internet]. Elsevier; 2020 [cited 2025 May 28];384:121306. https://doi.org/10.1016/J.JHAZMAT.2019.121306

4. Baz-Lomba JA, Di Ruscio F, Amador A, Reid M, Thomas K V. Assessing Alternative Population Size Proxies in a Wastewater Catchment Area Using Mobile Device Data. Environ Sci Technol [Internet]. American Chemical Society; 2019 [cited 2025 May 28];53:1994–2001. https://doi.org/10.1021/ACS.EST.8B05389

5. Laimou-Geraniou M, Nadarajan D, Bade R, Heath E. Spatiotemporal trends of new psychoactive substances in Slovenia through influent wastewater analysis. Journal of Hazardous Materials Advances [Internet]. Elsevier; 2025 [cited 2025 Jun 29];19:100781. https://doi.org/10.1016/J.HAZADV.2025.100781

6. Nadarajan D, Laimou-Geraniou M, Chappell A, Baduel C, Bijlsma L, Boogaerts T, et al. Wastewater analysis as a global toxicovigilance tool for the monitoring of new psychoactive substances. Water Res [Internet]. Pergamon; 2025 [cited 2025 Oct 23];124847. https://doi.org/10.1016/J.WATRES.2025.124847

7. Nadarajan D, O’Brien J, Cresswell S, Kele B, Mueller J, Bade R. Application of design of experiment for quantification of 71 new psychoactive substances in influent wastewater. Anal Chim Acta. Elsevier; 2024;1321:343036. https://doi.org/10.1016/J.ACA.2024.343036
